# Effect of *Schinus areira* L. Essential Oil on Attraction, Reproductive Behavior, and Survival of *Ceratitis capitata* Wiedemann

**DOI:** 10.3390/plants14050794

**Published:** 2025-03-04

**Authors:** Flavia Jofré Barud, María Pía Gomez, María Josefina Ruiz, Guillermo Bachmann, Diego Fernando Segura, María Teresa Vera, María Liza López

**Affiliations:** 1 Consejo Nacional de Investigaciones Científicas y Técnicas-CONICET, Argentina; jofrebarud@gmail.com (F.J.B.);; 2 Estación Experimental Agropecuaria, INTA San Juan, San Juan J5429XAB, Argentina; 3 Facultad de Agronomía, Zootecnia y Veterinaria, Universidad Nacional de Tucumán, San Miguel de Tucumán T4000, Argentina; 4 Instituto de Genética ‘Ewald A. Favret’, INTA, Grupo Vinculado al IABIMO (CONICET), Buenos Aires B1686, Argentina; 5 Facultad de Ciencias Agrarias y Veterinarias, Universidad del Salvador, Buenos Aires B1630AHU, Argentina

**Keywords:** pepper tree, essential oil, medfly, toxicity, attractant activity, oviposition, Y-tube olfactometer, sexual competitiveness, Mediterranean fruit fly

## Abstract

The essential oil (EO) of *Schinus areira* exhibits a chemical composition dominated by monoterpene and sesquiterpene hydrocarbons, with α-phellandrene, limonene, α-pinene, and p-cymene as major constituents. This study aimed to evaluate the effects of *S. areira* EO on the biology and behavior of the Mediterranean fruit fly, *Ceratitis capitata*, particularly its attraction to the EO and the impact on its reproductive behavior and survival. Females were attracted at the initial choice and the time spent in the arm of the Y-tube olfactometer with the EO was longer, while males were attracted at the final choice, indicating the attractive potential of *S. areira* EO for both sexes of *C. capitata*. Within the context of the sterile insect technique (SIT), the better performance of released sterile males allows more copulations with wild females in competition with wild males, increasing the efficacy of the SIT. Exposure of *tsl* sterile males to the EO did not enhance their sexual competitiveness and increased latency to initiate copulation, indicating potential adverse effects. In addition, in oviposition assays, only a low concentration of the EO stimulated egg-laying on treated substrates, possibly due to the absence of deterrent compounds such as linalool. Finally, the LD50 of the EO was <25 µg/fly for both females and males, at 72 h post-treatment. These findings highlight the potential of EOs as biopesticides that influence the behaviors of *C. capitata* and emphasize the need for further studies to optimize their application in integrated pest management strategies, including the SIT.

## 1. Introduction

*Ceratitis capitata* Wiedemann (Diptera: Tephritidae), known as the Mediterranean fruit fly or medfly, is one of the most important pests of fruit and vegetables worldwide and is considered one of the most invasive fruit flies, having spread from Africa to most of the tropical and temperate regions of the world [1]. It is a quarantine species with international restrictions imposed by medfly-free countries [2]. The species attacks more than 600 different hosts [3]. The female lays eggs inside the pulp of the fruit and the larvae feed on it, causing rapid deterioration of the fruit. Fully grown larvae (third instar) stop feeding and leave the fruit, burrowing into the soil to pupate. Adults emerge from the ground, and are the main target of management strategies due to the cryptic feeding habits of the larval and pupal stages in the soil. Management strategies usually used include monitoring and mass trapping, which are based on attraction [4,5]; the sterile insect technique (SIT), which is based on mating and oviposition [6,7]; and insecticide treatments, which are based on mortality [8,9]. These methods are generally combined in integrated pest management (IPM) programs [10]. The success of these control methods depends largely on adult behaviors and survival.

Insect pest control confronts a world with a growing population that demands sustainable strategies for food production and food safety. Biopesticides appear to be a novel alternative to be integrated into IPM programs through their application in modern agricultural practices for managing crop yield losses due to pest infestation. In searching for new biopesticide alternatives, researchers are intensively studying essential oils (EOs) due to their recognized effects on insect behavior. EOs are known for their repellent, insecticidal, and antifeedant activities, which make them candidate biopesticides with low toxicity to mammals and no detrimental environmental effects [11]. EOs can have both negative and positive effects on the behavior of insects. Both properties make EOs candidates to be incorporated in the technological strategies of IPM, such as lure-and-kill techniques, SIT, natural insecticides, and oviposition lures or deterrents. Particularly, the SIT is one of the most effective and environmentally friendly methods to control the medfly. This method involves the mass rearing and sterilization of males of the target species, which are then released in large numbers in the areas affected by the pest. The overall purpose of the SIT is for sterile males to inseminate wild fertile females, leading to a progressive decrease in the reproductive success of the population at the local site, hence diminishing population size [12]. Because the success of the SIT relies on inducing sterility in wild females, the ability of sterile males to attract and mate with wild females is essential. Thus, a higher mating success of sterile males impacts positively on SIT efficacy. Several EOs have been identified as long-range attractants that also enhance the mating success of *C. capitata*. These include EOs from hosts (orange, *Citrus aurantium* L.) [13] and non-hosts (ginger root, *Zingiber officinale* Roscoe, manuka, *Leptospermum scoparium* Forst and Forst, and tea tree, *Melaleuca alternifolia* (Maiden and Betche) Cheel) [14,15,16,17].

Several EOs also present as toxic to the medfly. EOs from orange and bitter orange are more toxic than the EO from lemon (*Citrus limon* (L.) Osbeck). The toxicity of orange and bitter orange EOs is similar to that of their major component limonene [18]. EOs from sandalwood, cedar, eucalyptus, lemongrass, and geranium are also toxic to newly emerged *C. capitata* adults [19]. In topical applications, EOs from *Rosmarinus officinalis* L., *Lavandula angustifolia* Miller, and *Thuja occidentalis* L. have good insecticidal activity at 24 h [20]. EOs from *Tagetes* species and *Mentha pulegium* L. have toxic properties as well [21,22].

*Schinus areira* L. (Anacardiaceae), commonly known as “aguaribay”, “pimientero”, or pepper tree, is native to South America. The fruits are used in culinary recipes as a substitute for pepper and the tree is used as a decorative plant in gardens. The leaves are used as an insect repellent and as a dye, and the bark exudate is used as an expectorant [23]. The EO of *S. areira* shows repellent, toxic, and feeding-deterrent properties against *Tribolium castaneum* (Coleoptera: Tenebrionidae) [24]. In addition, the EO from the leaves of *S. areira* shows insecticidal activity against *Rhipibruchus picturatus* (Coleoptera: Bruchinae) and inhibits the acetylcholinesterase enzyme in in vitro assays [25]. The Brazilian chemotype shows inhibitory activity against the bed bug *Cimex lectularius* [26], while *S. molle*, a close relative botanical species to *S. areira*, shows insect-repellent and insecticidal activity against *Trogoderma granarium*, *Tribolium castaneum* [27], and *Sitophilus oryzae* [28].

Based on the above, the aim of the present study was to analyze and test the bioactivity of the EO of *S. areira* on *C. capitata* adults through laboratory and semi-field assays. Our specific aim was to evaluate its attraction and toxicity to adults, its potential as a sexual enhancer for sterile males in the SIT, and its oviposition-deterrent effect on females.

## 2. Results

### 2.1. Composition of the Essential Oil of Schinus areira

The analysis of the EO of *S. areira* showed fifty-six compounds (97.5%). Monoterpenes represented 67.8% and sesquiterpenes represented 26.3% (Table 1). The main monoterpenes identified were α-phellandrene (18.8%), limonene (9.6%), β-phellandrene (7.3%), camphene (9.2%), α-pinene (5.6%), p-cymene (4.8%), and β-myrcene (4.4%), whereas the main sesquiterpenes identified were bicyclogermacrene (3.7%), δ-amorphene (4.4%), and β-caryophyllene (1.9%). All the compounds identified, their percentages, and retention times are summarized in Table A1.

### 2.2. Behavioral Responses

*Ceratitis capitata* adults responded positively to the air flow in the Y-tube olfactometer. A percentage (ca. 20–30%) of the adults tested remained in the central arm of the Y-tube for unknown reasons, whereas >70% made a choice between the control (acetone) and the EO. When males were exposed to the EO of *S. areira*, a preference was observed only in the final choice (χ^2^ = 4.75; *p* = 0.03). At the beginning of the trial, males showed a trend toward choosing the EO-treated arm, although no statistical differences were found (χ^2^ = 2.46; *p* > 0.05), whereas females showed a preference for the EO-treated arm (χ^2^ = 4.17; *p* = 0.04) (Table 2), but no significant differences were observed at the end of the trial (χ^2^ =3.11; *p* > 0.05). The time spent in the EO-treated arm was longer for both males and females. Males spent 1.3 times longer in the EO-treated arm than on the control arm; however, no significant differences were found regarding their preference for the EO (T^2^ = 3.18; *p* > 0.05). Females were attracted to the EO (T2 = 4.31; *p* = 0.040), spending 1.6 times longer in the EO-treated arm than in the control arm (Table 2).

### 2.3. Mating

#### 2.3.1. Mating Success

**Figure 1:**
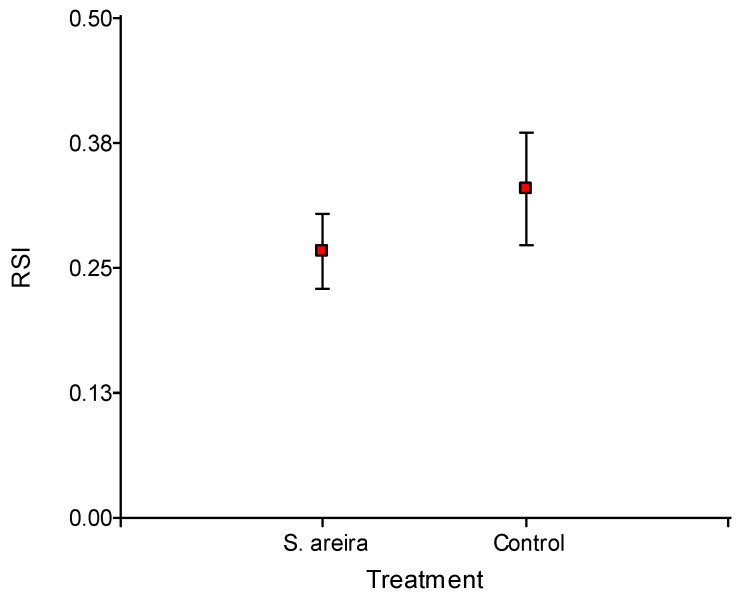
Relative sterility index (RSI) of *tsl* sterile males of *Ceratitis capitata* exposed and non-exposed (control) to the essential oil of *Schinus areira*.

The exposure to the EO volatiles had no effect on the mating success of *tsl* sterile males, as shown by the comparison of the relative sterility index (RSI) values between exposed and non-exposed males (Figure 1; GLM: F = 2.89 *p* = 0.1083). The *tsl* males exposed to *S. areira* EO had the same performance as control males (non-exposed *tsl* males).

#### 2.3.2. Latency to Mate

Comparison of the latency to mate between *tsl* sterile and wild males showed statistically significant differences when *tsl* males were exposed to *S. areira* EO. Wild males mated sooner than *tsl* males (W = 695.5; *p* = 0.0418) (Table 3). The latency to mate of *tsl* males exposed to *S. areira* EO was 1.68 times higher than that of non-exposed *tsl* males (control).

#### 2.3.3. Copula Duration

Comparison of the copula duration between *tsl* sterile and wild males revealed that copulations involving *tsl* sterile males were significantly shorter than those involving wild males in all cases (*S. areira*: W = 191.5, *p* = 0.0127; non-exposed: W = 179.5, *p* = 0.0001) (Table 4).

#### 2.3.4. Mating Location

Matings occurred mostly in the higher part of the trees, particularly on the inferior side of the leaves (Table 5 and Table 6). The control treatment showed significant differences in the location of mating pairs at different heights (high, medium, or low) (χ^2^ = 6.61; *p* = 0.0366) and leaf side (χ^2^ = 11.49: *p* = 0.0007) (Table 6).

### 2.4. Oviposition on Superficially Treated Grapes

**Figure 2:**
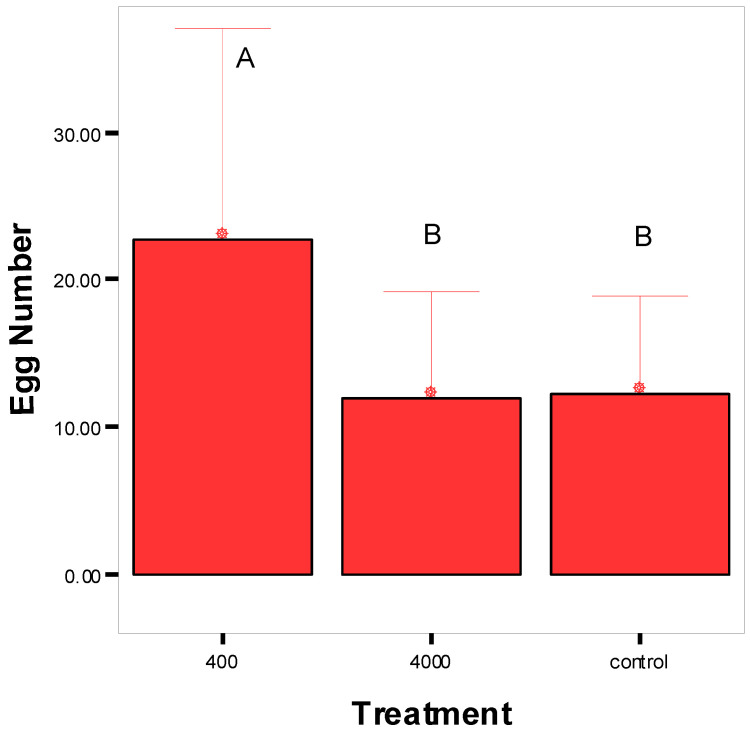
Number of eggs laid by females of *Ceratitis capitata* on grapes under different concentration treatments of *Schinus areira* essential oil. Different letters indicate significant differences at *p* < 0.05.

The infestation percentage observed on grapes superficially treated with 0 (control), 400, and 4000 ppm concentrations of *S. areira* EO was 55%, 80%, and 65%, respectively. Significant differences were observed in the number of eggs laid by females among the treatments (F = 45.5; d.f. = 2; *p* = 0.0001). Females laid more eggs on grapes treated with a low concentration of the *S. areira* EO (Figure 2).

### 2.5. Survival Response

A dose of 50 μg/fly caused 61.66 and 73.33% mortality of males and females, respectively, within 24 h post-treatment, whereas a dose of 100 μg/fly caused >90% mortality in both males and females at 24 h post-treatment. The mean lethal dose (LD50) of the oils was 21.83 and 24.84 µg/fly for females and males, respectively, at 72 h post-treatment (Table 7).

## 3. Materials and Methods

**Figure 3:**
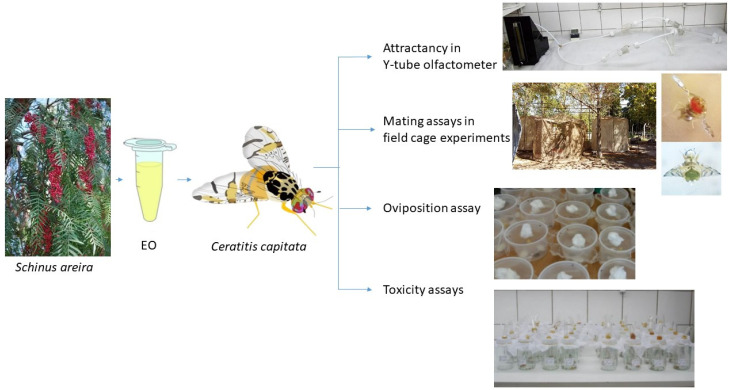
Overview of the assays performed to evaluate the effects of *Schinus areira* EO on attraction, mating, oviposition, and survival of *Ceratitis capitata* adults.

An overview of the methodology is presented in Figure 3.

### 3.1. Insects

Wild flies were obtained from different infested fruits, mainly peach (*Prunus persica* L.), plum (*Prunus domestica* L.), and fig (*Ficus carica* L.), collected in fruit orchards in San Juan, Argentina. In the laboratory, the fruit was placed on sawdust, which acted as a pupation substrate for *C. capitata* larvae emerging from the fruit. Adults were separated by sex within 48 h of emergence and maintained with water and food (hydrolyzed yeast and sugar, 1:3 proportion) ad libitum under controlled conditions of temperature (24 ± 2 °C), relative humidity (RH, 50 ± 5%), and light (12 h L/12 h D). Cohorts were kept apart.

Sterile males of *C. capitata* (*tsl* strain—Vienna8) were supplied by “Bioplanta de Insectos esteriles from Dirección de Sanidad Vegetal Animal y Alimentos, DSVAA”, Government of San Juan, Argentina, at the pupal stage (two days before adult emergence) after irradiation with Cobalt 60 at 120 Gy.

### 3.2. EO Production and Analysis

Plant material of *S. areira* was collected in the locality of Ullum, province of San Juan, Argentina (−31°26′30″ S, −68°39′38″ W). Fresh aerial parts were subjected to steam distillation using a Clevenger-type apparatus for 2 h. The EO was stored in micro-tubes at −18 °C until chemical analysis and its use in bioassays. The chemical analysis was performed by gas chromatography coupled to mass spectrometry (GC–MS). Mass spectra were obtained on a PerkinElmer Clarus 600 mass spectrometer, coupled directly to a PerkinElmer Series Clarus 600 gas chromatograph fitted with a DB-5 MS column (60 m long, 0.25 mm i.d., 0.25 µm film thickness). The GC–MS was operated under the following conditions: injector and detector temperatures of 250 °C; oven temperature programmed isothermal at 60 °C for 5 min, subsequently increased at 5 °C/min to 300 °C, and then held isothermally for 5 min. The carrier gas was helium, and the ionization voltage was 70 eV. The compounds were identified by the comparison of their retention index with reference to a homologous series of n-alkanes (C9–C25), by the comparison of their mass spectra with those reported in the literature [29], and by computer matching with the Wiley 8 and Adams libraries [30]. Relative percentage amounts were obtained directly from GC peak areas.

### 3.3. Behavioral Responses

The behavioral response of wild males and females was tested in a Y-tube olfactometer under laboratory conditions (24 ± 2 °C, 50 ± 5% RH). The glass Y-olfactometer (5 cm id, central arm 16 cm, lateral arms 10 cm) terminated in threaded-glass joints and Teflon screw caps that were connected to two separate glass vials (250 mL) with Teflon tubing. The Y-tube was held in an inclining position (angle 25° between the Y-tube and horizontal plane) by a tripod. An electric pump was used to pump air into the Y-tube olfactometer at 120 mL/min. The flux was controlled with a flow meter (Supelco). The entering air was moistened and pre-filtered through activated charcoal. The tested material consisted of 1 cm^2^ of filter paper treated with 2 µL of acetone containing 5 µg of EO compared with 2 µL of acetone for the control. Filter papers were placed in each vial and discarded after every single trial. For each trial, one fly was introduced into the central arm of the Y-tube and allowed to explore the tube with no air flow for 2 min. Then, the air stream was activated for 2 min. The first and final choices and the time each fly spent in each arm of the Y-tube olfactometer were recorded. Control experiments using air vs. air and solvent vs. solvent indicated that each arm of the Y-tube was equally visited (*p* < 0.05). Flies were 8–16 days old when tested. Twenty insects, ten males and ten females, were evaluated from 09:00 a.m. to 13:00 p.m. each day. A total of 140 females and 141 males were evaluated. The Y-tube olfactometer was cleaned after testing every 10 flies of one sex, before switching to the other sex. After testing five insects, the entire set-up, i.e., all parts of the Y-tube and vials, were rotated 180° to avoid position effects. The response of males and females to the EO was analyzed by means of a generalized linear model (GLM). For this, the proportion of individuals that made a choice in relation to the total individuals tested was analyzed using a binomial distribution of the error (logit link function). Initial and final choices were analyzed by the chi-square goodness-of-fit test. Data of permanence time were analyzed with the Friedman test to compare the control group with the EO group. In all cases, a significance level of 5% was used. The statistical program Infostat professional version 2017 was used [31].

### 3.4. Mating Assay of Sterile Males

The effect of the *S. areira* EO as a sexual enhancer of sterile males of *C. capitata* was assessed through direct sexual competition tests between sterile, mass-reared males and wild males for wild females. The female mating choice was evaluated in walk-in cages (see below) [32]. Emerged adults were kept under conditions of controlled temperature and humidity (24 °C, 50% RH) in environmental chambers, and provided water and an artificial diet (sugar and yeast hydrolysate, 12:1) ad libitum. Wild males and females were obtained as described above.

One day before initiating the mating assay, mass-reared males (4 days old) were exposed to the volatiles of the EO. Eighty sterile males were placed into a glass jar (3 L) with a polypropylene microtube containing 20 µL of the *S. areira* EO. The glass jar was covered with a nylon mesh. Exposure lasted 1.5 h. During exposure, flies were deprived of food and water. After exposure, the EO was removed, and food and water were restored. Non-exposed males were treated in the same way, but the glass jar had no EO. EO volatile exposure was conducted in isolated rooms for each treatment (exposed and non-exposed males) to avoid volatile contamination. Exposed and non-exposed males were labeled by providing them water colored with a food colorant (Circe). Different colors identified male treatments, and the colors were alternated between successive trials.

Field cage tests were conducted in walk-in cages (2.0 m in height, 3.0 m in diameter) located within the experimental field of the mass-rearing facilities of the Dirección de Sanidad Vegetal Animal y Alimentos (DSVAA), Government of San Juan, Argentina. Within the cages, three potted lemon trees (*Citrus limon* L.) approximately 2 years old were arranged to serve as mating arenas. During the tests, the temperature ranged from 18 °C to 26 °C and groups of 30 wild males, 30 wild females, and either 30 EO-exposed *tsl* sterile males or 30 non-exposed *tsl* sterile males were released inside each cage. Each cage with the 90 flies was considered a replicate. Males were released at 9:00 and left to acclimatize for 15 min, after which females were released. Once both sexes were inside the cage, an observer recorded the occurrence of copulation. Each mating pair was collected in vials and the number of matings, male identity, copulation start time, copulation end time, and mating location (cage or tree; tree height: high, medium, or low, and position on the leaf: lower or upper side) were recorded. Mating trials lasted until 13:00 and flies that did not mate were removed from the cage. Two replicates per treatment were carried out each day over a total of 3 days, thus obtaining six replicates (cages) per treatment in total. Repetitions with less than 20% of matings were discarded for data analysis. The effect of volatile exposure on male mating success was evaluated by comparing the number of matings achieved by *tsl* sterile males between each treatment by means of a GLM. The number of matings achieved by the *tsl* sterile males was the response variable, the treatment was the fixed factor, and the day was included in the model as a random factor. The error distribution was fitted to a binomial family with a logit link function. In addition, for each repetition, the RSI [33] was estimated following the guidelines of the Manual for Product Quality Control and Shipping Procedures for Sterile Mass-Reared Tephritid Fruit Flies [32]:

RSI = SW/(SW + WW)(1)

where SW are the matings achieved by sterile males and WW are the matings achieved by wild males. RSI values can vary from 0 to 1, where 0 indicates that all the females mated with wild males, 1 indicates that they all mated with sterile males, and 0.5 indicates that the number of matings achieved was equal for both types of males, so *tsl* sterile males are equally competitive with wild ones. Differences in the time it took the female to choose a male (latency to mate) and copula duration between *tsl* sterile and wild males were analyzed by means of a Wilcoxon test. The effect of volatile exposure on the location of mating couples (tree or cage, and position height) for both *tsl* sterile and wild males was analyzed by means of a χ^2^ test of independence, taking each treatment into account. Statistical analyses were conducted with Infostat professional version 2017 [31].

### 3.5. Oviposition on Superficially Treated Grapes

The influence of *S. areira* EO on the oviposition behavior of wild *C. capitata* was determined with an oviposition assay in grapes (*Vitis vinifera* L., Red Globe variety). A no-choice assay was conducted. Three days before the assays, three females and three males (7–9 days old) were placed in 1 L containers with water and food (yeast hydrolysate and sugar, 1:3) ad libitum, allowing the individuals to copulate. Grapes were used as oviposition substrate. The grapes were treated with solutions of different concentrations of *S. areira* EO. The solutions were prepared by dissolving the EO in 1 mL of acetone and then diluted with distilled water until the desired concentrations were achieved. The concentrations used were as follows: 0 (control), 400, and 4000 ppm in volume. Groups of 20 grapes were immersed in the solutions for 1 min, after which they were removed and placed on a glass tray to allow excess solution to evaporate from the surface. Depending on the concentration, one grape was placed in each container. After 48 h, the grapes were removed. The grapes were observed individually under a stereomicroscope (Leica) and the number of eggs laid was recorded. Ten replicates per treatment per day were performed over two days. Data were analyzed using a GLM that considered the number of eggs as the response variable, the concentration as the fixed effect, and repetition (day) as a random effect. The error distribution was fitted to a binomial family with a logit link function. The percentage of infestation was determined as the proportion of grapes on which eggs were detected relative to the total number of grapes. Statistical analysis was conducted with Infostat professional version 2017 [31].

### 3.6. Survival Response

The insecticidal activity of the EO was evaluated by topical application on *C. capitata* wild adults according to Jofre Barud et al. [34]. Flies (3–5 days old) were randomly selected and anesthetized under a nitrogen stream for 5 min. The immobilized flies were individually picked up and topical applications were performed with a volume of 2 µL/insect by means of an automatic micropipette for each dose on the dorsal side of the thorax. The doses used were 0, 10, 25, 50, and 100 µg/insect. All doses were prepared from fresh stock solutions obtained by dissolving the EO in 1 mL of acetone. Insects in the control group were treated with acetone alone. Both sexes were tested separately. Ten flies were tested by repetition, and three repetitions were used. The experiment was replicated two times. Mortality was recorded at 24, 48, and 72 h of treatment. To determine the LD50, probit analysis was conducted on mortality data at 72 h. The significance of the model was determined by a goodness-of-fit χ-square test estimated with maximum likelihood. Differences between LC50 values were considered significant when the respective 95% confidence intervals (CI95%) did not overlap. Data were analyzed with the statistical software SPSS 15.0 [35].

## 4. Discussion

*Schinus areira* is a South American tree known for its biological properties on different organisms, including its insecticidal activity on many insects. Here, the exposure of *tsl* sterile males of *C. capitata* to *S. areira* EO did not show an enhancement in the sexual competitiveness, but induced longer latency times to initiate mating and shorter copulations. However, such biological properties were demonstrated by causing the attraction of males and females in tests with an olfactometer, which demonstrated attraction of this fly to the volatiles of *S. areira*. Furthermore, the results show that concentrations of the order of 400 ppm of this EO on the surface of a natural oviposition substrate induced an increase in the number of eggs laid, but also that concentrations of one order of magnitude higher induced a decrease. The biological properties of the *S. areira* EO on *C. capitata* were further demonstrated when a relatively high toxicity for this EO was observed. All these biological properties of the *S. areira* EO on *C. capitata* could be used in pest management programs.

EOs exhibit a variety of effects on insect behavior, and their complex composition represents an advantage as biopesticides. One of their key benefits is the synergistic interaction between components, which helps prevent resistance development. The likelihood of insects evolving resistance to multiple molecular targets is significantly lower than to single-compound formulations. It is generally accepted that the main components of EOs drive their biological activity, while the magnitude of these effects depends on both the concentration of the principal compounds and the modulatory role of minor components [36].

The main chemical groups found in the *S. areira* EO in the present study showed a predominance of monoterpene and sesquiterpene hydrocarbons. This is in accordance with that found by Bigliani et al. [29]. These authors also identified limonene, camphene, α-pinene, and α-phellandrene as the main constituents in the *S. areira* EO. However, the main compound in our study was α-phellandrene, while the main compound found by Bigliani et al. [29] was α-pinene. Variation in EO composition is commonly related to genetic, climatic, and soil conditions. Consequently, different chemotypes can be found for the EO composition of a given botanical species. Other studies on the *S. areira* EO in Argentina have reported sabinene, bicyclogermacrene, and E-citral as main compounds [37] and the sesquiterpenoid alcohol 1-epi-cadinol, followed by δ-cadinene, alloaromadendrene, β-pinene, β-caryophyllene, and γ-cadinene [38]. The components of EOs largely explain their biological activities. Mono- and sesquiterpenes, the main components of EOs, show a variety of insecticidal and behavioral effects [39,40,41,42]. Given the variability in *S. areira* EO chemotypes, different effects on *C. capitata* can be expected. However, chemotypes rich in monoterpenes are likely to have the greatest impact on insects [43].

Host plant recognition through olfactory cues may involve species-specific compounds or specific interactions among ubiquitous compounds [44,45]. This process often exhibits redundancy in the composition of mixtures identified as hosts, as certain compounds can be substituted for others [46]. These characteristics highlight the potential of investigating volatile compounds capable of influencing pest insect behavior. Casaña-Giner et al. [47] evaluated the attraction of a group of 50 compounds, and found that one of the monoterpenes that attracted significantly more females than males of *C. capitata* was p-cymene. The fact that this compound was detected in *S. areira* EO could partially explain the positive response of females to this EO in the attraction assay. Furthermore, Jofre Barud et al. [34] reported that the EO of *Baccharis spartioides* significantly attracted females of *C. capitata*, with p-cymene identified as a component of the EO. Additionally, Hernández-Sánchez et al. [48] evaluated the attractiveness of some airborne terpenes of mango, also a host of *C. capitata*, and found that p-cymene and limonene attracted both males and females. Thus, these constituents might be responsible for the attraction response of *C. capitata* females to the *S. areira* EO at the dose tested. A percentage (ca. 20–30%) of the *C. capitata* adults remained in the central arm of the Y-tube olfactometer without making a choice. Although the exact reasons for this behavior remain unknown, it may have been likely influenced by the physiological state and motivation of each individual. Insect populations, including those of *C. capitata*, exhibit natural variability in behavioral responses, which can be attributed to differences in age, nutritional status, reproductive stage, and prior experiences. Flies that are not sufficiently motivated, due to factors such as recent feeding, lack of sexual receptivity, or stress, may fail to engage in active olfactory-driven movement. This diversity in individual responses highlights the complexity of insect behavior and suggests that non-responders represent a biologically relevant fraction of the population.

Some EOs improve the sexual performance of sterile males of *C. capitata*. However, results of the present study showed that exposure to *S*. *areira* EO did not show an enhancement in the sexual competitiveness of *tsl* sterile males of *C. capitata*. This EO could even be considered detrimental, as results showed a tendency to decrease the RSI, although no significant differences were observed. The RSI of control and exposed males was within that expected for *tsl* males of *C. capitata*, since less than 0.20 in a cage with a 1:1 (M:F) ratio is a cause for concern regarding male competitiveness [32]. Studies regarding the enhancement of sexual competitiveness of sterile males exposed to EOs of some host and non-host species have shown that the RSI is increased as compared to non-exposed males [33,34,49,50,51]. Whether or not the enhancing effect of some EOs on sexual competitiveness in sterile males of *C. capitata* is related to the EO composition remains unclear. Some studies have linked the presence of α-copaene to the enhanced sexual competitiveness and increased matings achieved by males exposed to EOs such as ginger root oil (GRO), manuka oil [52], angelica oil [49], and orange oil [15,51,53]. Even *Schinus polygama*, a relative species of *S. areira* that also improves sexual competitiveness, shows α-copaene in its EO composition. However, *B. spartioides* EO has shown an increase in matings and RSI, despite the absence of α-copaene in its composition [33]. Here, males did not show an increase in their sexual competitiveness after exposure to the *S. areira* EO, despite the EO containing α-copaene in its composition. Thus, other mechanisms not directly linked to the presence of α-copaene must be involved in the enhancement of sexual competitiveness of sterile males by some EOs. It is notable that *tsl* sterile males exposed to *S. areira* EO experienced an increase of 1.68-fold in their latency to mate, while wild males showed the shortest latency. Copulation required less time in *tsl* males than in wild males, irrespective of the exposure condition. The copula duration of sterile males exposed to *S. polygama* EO and GRO treatments has been found to be equal to that of wild males [33,50]. Previous studies have shown that exposure of sterile males to GRO did not affect copulation duration [50] and even reduced female remating rates [54]. In the present study, we found significant differences between *tsl* sterile and wild males regarding the copula location. Copulations of wild males were more frequently found on trees, while copulations of *tsl* sterile males were found equally on trees and in cages, irrespective of the exposure condition. Regarding the height location of matings, both wild and *tsl* sterile males were found frequently in the high parts of the cage or tree, and EO exposure seemed to not affect this pattern. Similarly, matings were seen more frequently on the inferior side of the leaf, irrespective of the exposure condition. These preferences are consistent with the species.

The factors influencing the oviposition site selection of certain fruit fly species include olfactory cues emitted by the host plant, as well as various physical and chemical characteristics of the fruit, such as size, shape, color, and the presence of specific chemical compounds [55]. During the contact phase, the decision of an insect to accept or reject an oviposition site is governed by the plant’s physical and chemical traits. Thus, in the present study, oviposition trials were conducted using a natural substrate (grapes) that was superficially treated with different concentrations of the *S. areira* EO. The results showed that only for the “low concentration” treatment (400 ppm) did females lay 1.8 times more eggs on grapes than the control. In this sense, Ioannou et al. [56] found that female ovipositional response to sweet orange EO is dose-dependent. Additionally, limonene, the most abundant chemical in all citrus oils, stimulates oviposition, whereas linalool, a representative compound of immature citrus fruit associated with high toxicity against immature stages of fruit flies, has a significant deterrent effect. In *S. areira* EO, limonene was a constituent present, while linalool was not identified in the oil. Hence, the low oviposition observed at high concentrations of the *S. areira* EO must involve other chemical compounds with deterrent activity. One possible compound with reported repellent activity is α-phellandrene, which has been tested against *Stomoxys calcitrans* [57] and *Bemisia tabaci* [58]. This compound was the main constituent in the *S. areira* EO and might be responsible for the low oviposition in the high-concentration treatment. The development of an effective egg trap based on an oviposition substrate treated with *S. areira* EO could serve as a powerful monitoring tool for gravid females. This would enable the measurement of the level of sterility induced in the population under SIT pressure, thus helping to assess the success of its application [59]. On the other hand, a spray treatment containing repellent constituents might deter *C. capitata* females from ovipositing on fruits, making it an effective control strategy.

EOs from aromatic species have toxic properties against *C. capitata* adults by topical application [19,20,21,34,36,60,61]. Monoterpenoids were the first inhibitors from plants which were considered to have anticholinesterase properties, partly from studies on chemical interactions between plant volatiles and insects [62,63,64]. Although the LD50 of *S. areira* EO was one order of magnitude higher than that reported for Spinosad, a biopesticide used to control *C. capitata* (LD50: 2693.14 ng/fly for females and 813.51 ng/fly for males at 24 h), synergistic effects between *S. areira* EO and Spinosad may represent a viable strategy. Such combinations could facilitate the inclusion of a natural product in pest management while simultaneously reducing the required concentration of the commercial insecticide, thereby lowering the environmental impact and minimizing the risk of resistance development [65]. The main component of *S. areira* EO was found to be α-phellandrene at 18.8%. This compound has been reported to be toxic at 27.56 µg/insect on *Tenebrio molitor* (Coleoptera: Tenebrionidae) adults after 48 h of exposure [66]. In addition, Jung et al. [67] found a LC50 of 0.28 mg/cm^2^ after 24 h of contact exposure against adult females of the German cockroach *Blattella germanica* (Blattodea: Blattellidae), while Ali et al. [68] found LC90 values of 19.3 ppm and 36.4 ppm against *Aedes aegypti* (Diptera: Culicidae) and *Anopheles quadrimaculatus* (Diptera: Culicidae), respectively. A study by Aboalhaija et al. [69] reported the anticholinergic and butyryl-cholinesterase properties of the EO and extracts from *S. molle* (Sapindales: Anacardiaceae), a close relative species to *S. areira*, and α-phellandrene was found as the main compound in the EO composition. Thus, the insecticidal properties of *S. areira* EO on *C. capitata* males and females might be related to the high content of α-phellandrene in the oil. The findings of this study highlight the potential of *S. areira* EO as a promising biopesticide, combining its toxic properties and attractant effects to target *C. capitata*. The high content of α-phellandrene in *S. areira* EO is likely responsible for its significant insecticidal activity, while the presence of compounds such as p-cymene and limonene demonstrate its ability to attract females, making it particularly effective for behavior-based control strategies. These dual properties suggest that *S. areira* EO could potentially be integrated into lure-and-kill techniques or egg-laying traps to enhance pest management programs. Further research should explore the optimization of EO concentrations and formulations to maximize its efficacy for application in environmentally sustainable pest-control solutions.

## Figures and Tables

**Table 1:** Main chemical groups and composition of the essential oil of *Schinus areira*.

Chemical Group	Area (%)
Aliphatic hydrocarbons	0.2
Monoterpene hydrocarbons	65.4
Oxygenated monoterpenes	2.2
Sesquiterpene hydrocarbons	18.8
Oxygenated sesquiterpenes	7.3
Not identified	3.6
Total	97.5

**Table 2:** Behavioral responses of females and males of *Ceratitis capitata* in the Y-olfactometer to volatiles of the essential oils of *Schinus areira* vs. a solvent control (acetone) (N = 141 ♀; 140 ♂).

Sex	Variable	Y-Tube Arm		Estimator ^a,b^	*p* ^c^
		EO	Control		
Females	Initial Choice *	58	38	4.17	0.040 *
	Final Choice ^≠^	55	38	3.11	0.080
	Time Spent (s)	68.8	43	4.31	0.040 *
Males	Initial Choice	60	44	2.46	0.12
	Final Choice	62	40	4.75	0.03 *
	Time Spent (s)	60.7	46.8	3.18	0.077

Different letters among the treatments in the time spent in each arm denote significant differences at a *p* = 0.05 level according to the Wilcoxon signed-paired-rank test. ^(a)^ Estimator for the first and final choices: chi-square χ^2^, ^(b)^ estimator for time spent in each arm of the Y-olfactometer: T^2^, ^(c)^ *p*-value. * Initial choice: first choice made by each fly between the EO-treated arm and the control arm at the beginning of the trial. ^≠^ Final choice: last choice made by each fly between the EO-treated arm and the control arm at the end of the trial.

**Table 3:** Latency to mate of *tsl* sterile males exposed and non-exposed (control) to the *Schinus areira* essential oil and wild males of *Ceratitis capitata*.

	*Tsl* Males		Wild Males		W	*p*
	Median	Q1–Q3	Median	Q1–Q3		
*S. areira*	126	17–155	28	13–95	695.5	0.0418
Control	75	12–105	35	11–98	1190	0.7163

**Table 4:** Copula duration (min) for *tsl* sterile males exposed and non-exposed (control) to the essential oil of *Schinus areira* and wild males *Ceratitis capitata*.

	*Tsl* Males		Wild Males		W	*p*
	Median	Q1–Q3	Median	Q1–Q3		
*S. areira*	122	99–161	189	156–210	191.50	0.0127
Control	128	104–143	190	174–216	179.50	0.0001

**Table 5:** Number of matings achieved by *tsl* sterile males exposed and non-exposed (control) to the essential oil of *Schinus areira* and wild males of *Ceratitis capitata* in each location (cage vs. tree).

		Males		χ^2^ (d.f = 1)	*p*
Treatment	Location	*Tsl*	Wild		
*S. areira*	Tree	5	71	8.37	0.0038
	Cage	9	25		
Control	Tree	8	91	27.30	<0.0001
	Cage	11	9		

**Table 6:** Height location and leaf side of *Ceratitis capitata* mating couples in the field mating tests involving *tsl* males exposed and non-exposed (control) to *Schinus areira* essential oil, and wild males.

			Males		χ^2^ (d.f. Height = 1*_S.areira_*; 2_control_; d.f. Leaf Side = 1)	*p*
			*Tsl*	Wild		
*S. areira*	Height	High	15	95	0.63	0.4256
		Medium	0	4		
		Low	-	-		
	Leaf side	Inf	4	69	1.64	0.1997
		Sup	1	4		
Control	Height	High	15	92	6.61	0.0366
		Medium	3	8		
		Low	1	0		
	Leaf side	Inf	7	91	11.49	0.0007
		Sup	1	0		

**Table 7:** Toxicity of the essential oil of *Schinus areira* at 24, 48, and 72 h after topical application on *Ceratitis capitata* adults (N = 30).

Sex	Time (h)	LD_50_ µg/fly	Slope ± SE	χ^2^	df
Males ♂	24	30.65 (24.84–37.48)	2.05 ± 0.18	23.89	14
	48	27.34 (21.49–34.00)	1.94 ± 0.18	25.81	
	72	23.84 (17.98–30.20)	1.94 ± 0.18	28.30	
Females ♀	24	28.63 (23.00–35.09)	2.41 ± 0.19	31.43	14
	48	24.43 (19.78–29.44)	2.27 ± 0.19	24.18	
	72	21.83 (18.73–25.03)	2.19 ± 0.19	19.66	

df: degrees of freedom.

## Data Availability

The original contributions presented in the study are included in the article; further inquiries can be directed to the corresponding author.

## Abbreviations

The following abbreviations are used in this manuscript:

EOs	Essential oils
RSI	Ratio sterility index
SIT	Sterile insect technique
IPM	Integrated pest management
GC–MS	Gas chromatography–mass spectrometry
*tsl*	Thermo-sensitive lethal

## Appendix A

**Table A1:** The chemical composition of the essential oils obtained from *Schinus areira* and their relative proportions (% total area).

RI	Compound	Area (%)
839	2,4-Dimethyl-1-heptene	tr
927	Tricyclene	1.2
939	α-Pinene	5.6
956	Camphene	9.2
977	Sabinene	0.9
984	β-Pinene	3.7
992	β-Myrcene	4.4
1014	α-Phellandrene	18.8
1031	p-Cymene	4.8
1037	Limonene	9.6
1039	β-Phellandrene	7.3
1063	ɣ-Terpinene	tr
1090	Terpinolene	0.1
1108	Nonanal	0.2
1132	43 (99.9); 93 (48.8); 71 (43.3); 69 (42.0); 94 (38.1); 95 (36.0); 111 (35.8); 79 (35.2); 139 (34.7); 55 (34.4).	tr
1151	43 (99.9); 55 (75.0); 41 (72.4); 92 (68.8); 91 (63.7); 69 (61.1); 93 (51.4); 81 (48.6); 83 (46.4); 79 (42.1)	tr
1183	95 (99.9); 110 (36.2); 41 (24.2); 109 (23.7); 81 (21.2); 67 (18.5); 39 (12.6); 55 (11.5); 108 (11.2); 53 (93)	tr
1189	1-Terpinen-4-ol	0.3
1197	Cryptone	tr
1203	α-Terpineol	tr
1213	cis-Sabinol	tr
1254	43 (99.9); 97 (58.3); 107 (42.8); 41 (31.8); 55 (23.0); 39 (16.7); 79 (15.5); 69 (14.3); 109 (14.2); 53 (13.9)	0.4
1290	Bornyl acetate	1.9
1338	δ-Elemene	0.1
1353	α-Cubebene	tr
1383	α-Copaene	0.1
1396	β-Elemene	0.9
1417	α-Gurjunene	0.6
1432	β-Caryophyllene	1.9
1450	Aromadendrene	tr
1459	(cis)-Muurola-3,5-diene	0.1
1468	α-Humulene	0.6
1473	(cis)-Muurola-4(14),5-diene	0.3
1481	cadina-1(6),4-diene(trans)	0.1
1484	γ-Muurolene	0.4
1493	(trans)-Muurola-4(14),5-diene	1.4
1502	Viridiflorene	0.2
1507	Bicyclogermacrene	3.7
1524	γ-Cadinene	0.8
1528	δ-Amorphene	4.4
1532	(trans)Calamenene	0.7
1543	(trans)Cadina-1(2),4-diene	0.2
1547	α-Cadinene	0.2
1552	α-Calacorene	tr
1559	Elemol	2.2
1586	Ledol	0.3
1590	Germacrene D-4-ol	1.1
1592	Spathulenol	0.9
1617	Viridiflorol	0.5
1622	97 (99.9); 41 (97.3); 81 (79.6); 55 (77.7); 79 (75.1); 69 (73.6); 107 (63.1); 67 (54.4); 93 (53.5); 177 (46.7).	2.5
1647	Eudesmol (10-epi-gamma)	0.5
1661	Cadinol (epi-alpha)	0.5
1664	Muurolol (epi-alpha)	1.3
1671	α-Cadinol	1.2
1674	α-Eudesmol	0.8
1715	84 (99.9); 41 (66); 81 (65.7); 55 (50.8); 83 (44); 109 (43.2); 67 (41.1); 69 (39.9); 121 (29.9); 93 (29.2).	0.6

tr: trace compounds.
